# Progression of hepatocellular carcinoma after radiofrequency ablation: Current status of research

**DOI:** 10.3389/fonc.2022.1032746

**Published:** 2022-11-22

**Authors:** Shilun Wu, Zhuxin Li, Changyu Yao, Shuying Dong, Jun Gao, Shan Ke, Ruhang Zhu, Sen Huang, Shaohong Wang, Li Xu, Chen Ye, Jian Kong, Wenbing Sun

**Affiliations:** Department of Hepatobiliary Surgery, Beijing Chaoyang Hospital Affiliated to Capital Medical University, Beijing, China

**Keywords:** hepatocellular carcinoma, radiofrequency ablation, residual tumor, tumor progression, prevention

## Abstract

Hepatocellular carcinoma (HCC) remains an important disease for health care systems in view of its high morbidity, mortality, and increasing incidence worldwide. Radiofrequency ablation (RFA) is preferred to surgery as a local treatment for HCC because it is safer, less traumatic, less painful, better tolerated, causes fewer adverse reactions, and allows more rapid postoperative recovery. The biggest shortcoming of RFA when used to treat HCC is the high incidence of residual tumor, which is often attributed to the vascular thermal deposition effect, the wide infiltration zone of peripheral venules, and the distance between satellite foci and the main focus of the cancer. Recurrence and progression of the residual tumor is the most important determinant of the prognosis. Therefore, it is important to be aware of the risk of recurrence and to improve the efficacy of RFA. This review summarizes the relevant literature and the possible mechanisms involved in progression of HCC after RFA. Current studies have demonstrated that multimodal treatments which RFA combined with other anti-cancer approaches can prevent progression of HCC after RFA.

## 1 Introduction

Hepatocellular carcinoma (HCC) is the most common type of liver cancer and accounts for 90% of cases. It is associated with a poor prognosis because of a high recurrence rate and a lack of effective therapeutic options (1). Patients with early-stage HCC can be treated by liver transplantation, surgical resection, or radiofrequency ablation (RFA) as first-line therapies (2, 3). Although surgical resection and liver transplantation have been regarded as the optimum therapeutic strategies, their application is limited in many cases because of lack of a donor, decompensated liver function, and failure to meet specific (Milan) selection criteria (4).

RFA is a minimally invasive and repeatable treatment that is associated with limited procedure-related morbidity, which results in better cost-effectiveness and quality of life. RFA is also considered as an alternative for patients not eligible for surgical resection or liver transplantation and those awaiting liver transplantation. Many studies have demonstrated that the efficacy of RFA and surgical resection in terms of survival outcomes are similar for a single small HCC of ≤3 cm (5, 6). However, RFA appears to be inferior to surgical resection in terms of local control and disease-free survival (7). Rapid intrahepatic neoplastic progression and metastasis of HCC after RFA, indicating more aggressive biological behavior, has also been found in some clinical centers (8). The mechanisms underlying progression of HCC after RFA remain poorly understood, and it is important to develop targeted therapy that can improve the prognosis of this disease. This review discusses the literature on the potential mechanisms of progression of HCC after RFA in the hope of finding preventative strategies.

## 2 Tumor progression in HCC after RFA

**Figure 1:**
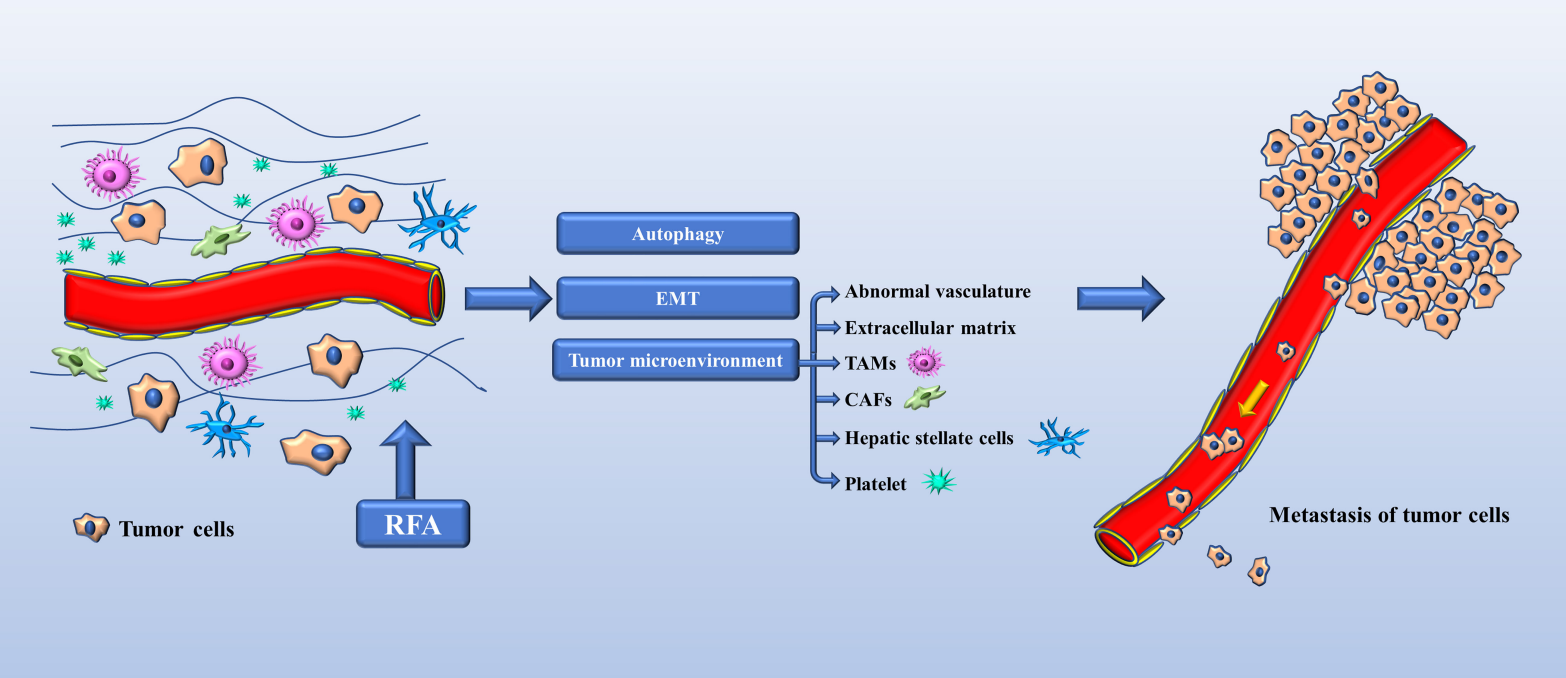
Mechanisms of tumor progression in HCC after RFA. EMT (epithelial-mesenchymal transition), TAMs (tumor-associated macrophages), CAFs (carcinoma-associated fibroblasts).

Since the first experimental hepatic RFA procedure was performed in 1990 (9), there has been extensive research on RFA, and it is now regarded as a curative treatment for HCC. However, many clinical centers have been reporting an increasing number of cases of progression of HCC after RFA. Seki et al. (10) described a patient who underwent transcatheter arterial chemoembolization and RFA for a small HCC measuring 2.5 cm, and enhanced magnetic resonance following treatment showed complete tumor necrosis and did not reveal any tumor around the treated area. However, numerous tumors around the treated area were observed on enhanced computed tomography 50 days after RFA. Koda et al. (11) reported a similar case involving well-differentiated HCC that was treated by RFA and reduced to 2.5 cm in diameter by 6 months after the procedure but rapidly enlarged to 6 cm in the next 2 months and progressed to lymph node metastasis. Autopsy findings showed both sarcomatoid and trabecular HCC cells. This was the first reported case of sarcomatous HCC after RFA. Portolani et al. (12) subsequently reported on three patients with small HCC treated with RFA, in whom imaging confirmed complete ablation. However, tumor regrowth was diagnosed at 3, 4, and 6 months after RFA and was associated with extensive liver and parietal wall involvement. Ruzzenente et al. (13) reported on 87 patients with cirrhosis and 104 HCCs that were treated by RFA. In 4 patients, although complete local necrosis was achieved, rapid intrahepatic neoplastic progression was observed following RFA. After 30 days, there was a rapid increase in alpha fetoprotein in three of these four patients, two of whom died as a result of disease progression after 2–3 months of follow up. Baldan et al. (14) reviewed 401 cases of HCC treated by RFA from 13 centers in Italy and identified tumor seeding in four patients and rapid unexpected disease progression on another 10 patients. Shiozawa et al. (15) investigated 1073 lesions in 538 patients who underwent ultrasound-guided RFA between April 1999 and March 2008 and documented rapid aggressive disease progression in 0.65% of cases. In a study of the perfusion features of local recurrence of HCC after RFA, Wu et al. (16) demonstrated that enhancement was more homogeneous, the border was more poorly defined, washout was more marked, and that there were fewer feeding vessels and areas of inner necrosis in the recurrent HCC than in the initial HCC. Moreover, the tumor stem cell markers CD133 and EpCAM were also both highly expressed in specimens from the patients with recurrent disease. As shown in **Figure 1**, we reviewed the relevant mechanisms.

## 3 Cause of residual tumor tissue

RFA of a tumor requires local application of extremely high temperature, which can cause irreversible cell injury and ultimately tumor apoptosis and coagulative necrosis. According to the size and shape of the needle tip, a spherical ablated area is generated in about 10–30 minutes, generally from 2 cm to 5 cm in diameter. With RFA, the zone of active tissue heating is limited to the few millimeters surrounding the active electrode, with the remainder of the ablation zone heated *via* thermal conduction. With an increase in the size of the target area, the efficacy of the treatment is reduced (17). Moreover, certain tissue properties, such as electrical conductivity, thermal conductivity, dielectric permittivity, heat capacity, and blood perfusion rate, have a substantial effect on the growth of ablation zones. Interestingly, a temperature >100°C is less effective because the desiccation that results at these temperatures, which manifests as water vapor and burnt tissue, increases the tissue impedance and therefore limits further electrical conduction through the remaining tissue (18).

Furthermore, a cytotoxic temperature is difficult to maintain if the ablated tumor is close to large blood vessels (19). This heat-sink effect is a commonly described limitation of RFA and occurs when heat that is absorbed by flowing blood or air is carried away from the area of ablation; in these cases, the lower energy intensity within the passive zone is not able to achieve thermally toxic temperatures in proximity to the cooling vasculature (20). Therefore, tumor tissue that is adjacent to the vasculature is less susceptible to thermal damage.

## 4 Mechanisms of tumor progression in HCC after RFA

### 4.1 Changes in biological behavior of HCC cells after RFA

RFA may directly change the proliferation, invasion, and metastasis of HCC cells. Obara et al. (21) assessed the proliferation rate, heat sensitivity, and invasive capacity of several HCC cell lines in response to heat treatment and demonstrated that even a single session of heat treatment could induce further transformation of these cells. Ke et al. (22) established a rabbit model of residual VX2 hepatoma after RFA and identified inadequate RFA caused by temperature that was too low at the target sites to be a potentially important cause of rapid disease progression. Rapid progression of residual hepatic VX2 carcinoma could be facilitated by overexpression of several molecular factors, such as proliferating cell nuclear antigen, matrix metalloproteinase 9, vascular endothelial growth factor (VEGF), hepatocyte growth factor, and interleukin (IL)-6. Zhang et al. (23) also demonstrated that RFA promoted proliferation, migration, and invasion of HepG2 and SMMC7721 cells. Epigenetic regulation also has an important role in maintaining homeostasis when cells are exposed to acute physicochemical stresses. Moreover, in a mechanistic study of the role of m6A machinery in recurrence of HCC after RFA, Su et al. (24) found that sublethal heat treatment increased m6A modification of the epidermal growth factor receptor (EGFR) in the vicinity of the 5’ UTR region and promoted its binding with YTHDF1, which enhanced the translation of EGFR mRNA and promoted viability and metastasis of HCC cells after RFA.

### 4.2 Autophagy

**Figure 2:**
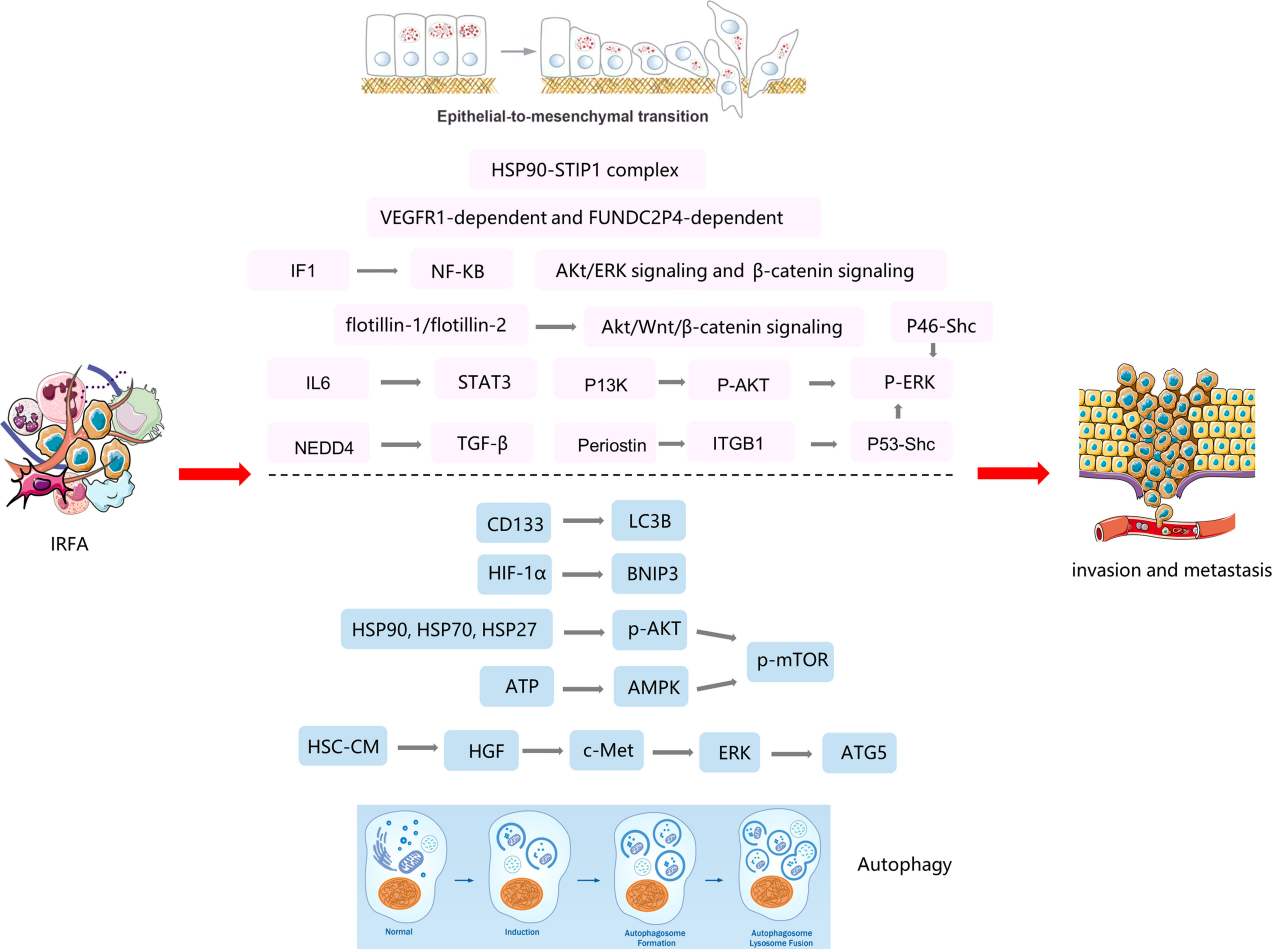
The mechanisms of EMT and autophagy in tumor progression after insufficient RFA(IRFA) of HCC.

Autophagy is evolutionarily conserved cellular process wherein components of cells are degraded by sequentially formed autophagic vesicles and is also a cellular process used by cancer cells to replicate under various adverse conditions, such as oxidative stress, endoplasmic reticulum stress, mitochondrial stress, and starvation. The possible signaling pathways of autophagy in HCC after RFA are listed in **Figure 2**. Wang et al. (25) reported that autophagy participated in the enhanced viability and invasion of HCC cells after inadequate RFA. They also found that CD133 became localized to autophagosomes and was suppressed by 3-MA or chloroquine, which could suppress RFA-induced cell viability, invasion, and autophagy. Zhao et al. (26) showed that insufficient RFA induced an anoxic microenvironment, autophagy, and autophagic flux in tumor cells, which have an important role in tumor relapse and proliferation. Furthermore, Xu et al. (27) demonstrated that insufficient RFA increased autophagy in residual HCC cells *via* the hypoxia-inducible factor 1 (HIF)-1α/BNIP3 pathway, which is involved in increased proliferation, migration, and invasion of tumor cells. Chen et al. (28) found that the heat shock protein90 (HSP90)/Akt/mTOR pathway is involved in the signaling between autophagy and HSPs after incomplete thermal ablation. And subsequent studies found that the HSP90 inhibitor 17-AAG, in combination with the autophagy inhibitor 3-mA, promoted hepatocellular carcinoma apoptosis following incomplete thermal ablation more significantly than monotherapy, suggesting an association between heat-induced heat shock processes and autophagy (29). Jiang et al. (30) showed that sublethal heat stress induced protective autophagy against heat-induced apoptosis in HCC *via* the ATP-AMPK-mTOR axis, and the inhibition of autophagy by CQ or siRNA targeting the autophagy-related genes Beclin-1 and Atg5 enhanced heat-induced apoptosis. Zhang et al. (31) found that activated hepatic stellate cells promote progression of residual HCC cells after sublethal heat treatment from autophagic survival to proliferation *via* HGF/c-Met signaling. In an animal model, inhibiting autophagy in combination with c-Met inhibitor significantly thwarted tumor progression of residual HCC after incomplete thermal ablation *via* the suppressed autophagy, the decreased proliferation and the increased apoptosis.

### 4.3 Epithelial-mesenchymal transition

Trans-differentiation of epithelial cells into motile mesenchymal cells, a process known as epithelial-mesenchymal transition (EMT), is activated in an aberrant manner under pathological conditions, including organ fibrosis and cancer (32). To acquire an invasive phenotype for metastatic progression in cancer, carcinoma cells exploit EMT to facilitate their dissociation from the primary tumor and dissemination into the circulation. EMT also endows tumor cells with enhanced stemness and increased resistance to immune clearance and various iatrogenic insults (33). The mechanisms of EMT in tumor progression after RFA of HCC are listed in **Figure 2**.

An increasing body of data suggests that EMT also participates in progression of HCC after RFA. Some studies have shown that P-ERK1/2 plays an important role in heat-induced EMT (34). Four studies (35–38) performed heat treatment on MHCC97H, HepG2, HuH7, and HEP3B cell lines and found that ERK was significantly phosphorylated. EMT was attenuated after inhibition of P-ERK1/2, which was similar to other ways of activating EMT. They found that P13K, P46-Shc, and Periostin were activated as upstream proteins of ERK after heat treatment, causing tumor invasion and metastasis. Flotillin-1 and flotillin-2 were found to be upregulated in HCCLM3 cells following heat treatment and in residual HCCLM3 xenograft cells after RFA, which altered the status of EMT and metastatic potential *via* activation of the Akt/Wnt/β-catenin signaling pathway (39). Zhang et al. (40) also found that incomplete RFA enhanced the invasive and metastatic potential of residual cancer, accompanying with EMT-like phenotype changes by activating β-catenin signaling in HCCLM3 cells. Subsequent study found that a combination of interferon alpha and an herbal compound known as “songyou yin” significantly weakened the enhanced metastatic potential of residual HCC after RFA *via* attenuation of EMT and that this effect was mediated by inhibition of activation of β-catenin (41). Su et al. (42) found that incomplete RFA induced the formation of Stress-induced phosphoprotein 1 (STIP1) - HSP90 complex, which mediated heat-induced EMT and metastasis in HCC cells. Tan et al. (43) showed that sublethal heat treatment increased the expression of cancer stem cell markers and markers of metastasis and promoted the ability of HCC cells to migrate after RFA. They also found that blockade of VEGFR1 could reduce heat-induced enhancement of migration and stemness. Kong et al. (44) found that ATPase inhibitory factor 1 (IF1) promoted EMT and angiogenesis in HCC after inadequate RFA, that this ability was markedly inhibited after IF1 knockdown, and that the sensitivity of HCC cells to sorafenib was attenuated after RFA *via* the nuclear factor kappa-B signaling pathway. Zeng et al. (45) reported that downregulation of lncRNA FUNDC2P4 promoted EMT, leading to proliferation, invasion, and migration of tumor cells by reducing expression of E-cadherin in residual HCC after RFA, which suggests that FUNDC2P4 may have value for prevention and treatment of recurrent HCC. Zhou et al. (46) demonstrated that insufficient ablation at a low temperature induced EMT and promoted tumor aggressiveness that was mediated by the IL-6/STAT3/Snail pathway. Targeting EMT could suppress tumor progression in HCC after RFA. Li et al. (47) showed that the upregulation of Nedd4 in HCC insufficient ablation tissues was induced by METTL14-mediated N6-methyladenosine modification after sublethal heat treatment. Besides, Nedd4 enhanced TGF-β/smad/EMT signal transduction by directly binding to TGFBR1 and forming K27-linked ubiquitin at Lysine 391 mediated HCC progression. Knockdown of Nedd4 inhibited HCC metastasis and growth *in vitro* and *in vivo*.

## 5 Tumor microenvironment

The tumor microenvironment in HCC is a complex and spatially structured mixture of hepatic non-parenchymal resident cells, tumor cells, immune cells, and tumor-associated fibroblasts. All these cell populations interact in a dynamic manner through cell-cell contact and release or recognition of cytokines and other soluble factors. This complex interplay between cells has a substantial influence on tumor immune evasion (48).

### 5.1 Abnormal vasculature

Rapid development of new vascular networks is required in tumors in order to support a high cell proliferation rate. These networks are different from those of normal blood vessels and are characterized by distorted and chaotic branches, heterogeneity of the vascular lumen, incomplete pericyte coverage, an abnormal basement membrane, increased vascular permeability, hypoxia, and increased tissue hydraulic pressure (49), which are crucial for metastasis and escape of cancer cells (50). Many studies have shown that RFA promotes angiogenesis in residual liver cancer tissue and worsens its abnormal vasculature.

#### 5.1.1 Tumor-associated endothelial cells

Tumor-associated endothelial cells (TAECs) form the inner layer of tumor blood vessels and are an important part of the tumor microenvironment. Unlike normal endothelial cells, TAECs show morphological and phenotypic abnormalities at the cellular and molecular levels. Furthermore, angiogenic ability and drug resistance have been shown to be significantly higher in TAECs than in normal endothelial cells (51). TAECs are constituents of blood vessels that provide oxygen and nutrients for tumor cells and act as gatekeepers that allow these cells to escape and enter the circulation (52). An imbalance of tumor endothelial cells leads to loss of normal vascular barrier function and provides a channel for metastasis of tumor cells (53). Kong et al. identified significant enhancement of the migration and tube formation ability of TAECs after RFA, which may play a key role in the rapid growth of residual HCC. TAECs could also increase the invasive ability of HCC cells by secreting a variety of cytokines, including IL-8, IL-6, monocyte chemoattractant protein-1, and Gro-α. Furthermore, expression of E-selectin, intercellular adhesion molecule 1 (ICAM-1), and vascular cell adhesion molecule 1 was found to be upregulated in TAECs after insufficient RFA, suggesting that upregulation of adhesion molecules may be one of the mechanisms of the enhanced adhesion between TAECs and HCC cells. Angiogenic capability and drug resistance was also found to be higher in TAECs than in normal endothelial cells (54).

#### 5.1.2 Angiogenesis

Angiogenesis is the term used to describe the formation of new blood vessels in already existing vasculature. This process is the result of the synergistic action of tumor cells and the tumor stroma and is a prerequisite for metastasis (55). Folkman first proposed that tumor growth and metastasis depend on angiogenesis in 1971 (56). The mechanisms that drive angiogenesis are complex and have an important role in progression of HCC after insufficient RFA. VEGF plays an important part in angiogenesis; its most important member is VEGFA, which can directly stimulate the movement, proliferation, and division of vascular endothelial cells and increase the permeability of the microvasculature. Ke et al. (22) found that inadequate RFA caused by a low target temperature resulted in a significant increase in expression of VEGF in residual tumor tissue and promoted metastasis of liver cancer. Liu et al. (57) also showed that RFA promoted growth of residual HCC by increasing expression of VEGF *via* activation of CaMKII-induced ERK. Ahmed et al. (58) found that RFA of normal liver tissue stimulated tumor growth in distant subcutaneous tissue, which was mediated *via* the hepatocyte growth factor/c-Met pathway and activation of VEGF and that this process could be suppressed by inhibition of VEGF.

Tumors with a high cell proliferation rate and undergoing active growth have a significantly reduced oxygen supply, especially cells in the core of the tumor, and activation of HIF-1 promotes the release of more pro-angiogenic factors, especially VEGFA, from tumor cells and stromal cells (59). Kong et al. (60) found that RFA could promote angiogenesis in residual HCC *via* HIF-1a/VEGFA and that the HIF-1a inhibitor YC-1 reversed this process. Xu et al. (61) demonstrated that hypoxia and hypoxia-driven angiogenesis have an important role in the recurrence of HCC after RFA and that sorafenib is an effective inhibitor of the HIF-1a/VEGFA pathway.

#### 5.1.3 Vascular permeability

Increased vascular permeability results mainly from the loss of connexin between endothelial cells and causes destruction of the integrity of the vascular barrier, which in turn affects the ability of tumor cells to cross the vascular barrier. Endothelial cells are connected by connective proteins, including adhesion connexins, such as VE-cadherin and catenins, and by tight junctions, such as ZO-1 and claudin-5. Studies have confirmed that loss of endothelial intercellular connexin can promote permeability of the tumor vasculature and metastasis (62). Kong et al. (63) found that ICAM-1 induces aggregation and activation of platelets, increases endothelial permeability *via* Ezrin/VE-cadherin, and promotes tumor migration across endothelial cells in HCC after insufficient RFA.

#### 5.1.4 Vasculogenic mimicry

Vasculogenic mimicry (VM) is different from the classical tumor angiogenesis pathway, independent of endothelial cells, and involves hollow lumens composed of basement membrane and peripheral cancer cells (64). VM has been discovered to be a method of angiogenesis in many malignant tumors and provides a novel strategy for the clinical treatment of angiogenesis in tumors, which is related to the invasion, metastasis, and poor prognosis of HCC. Cancer stem cells and EMT participate in VM (65). Jia et al. (66) found that platelet lysates in patients with HCC after RFA can promote EMT and activation of Akt, ERK1/2 and Smad3 signals, further promoting tumor VM and metastasis of HCC after RFA. Kong et al. (44) also demonstrated that EMT participated in VM and promoted progression of HCC after insufficient RFA.

### 5.2 Extracellular matrix

The extracellular matrix (ECM) is a non-cellular three-dimensional macromolecular network composed of collagens, proteoglycans/glycosaminoglycans, elastin, fibronectin, laminins, and several other glycoproteins. The ECM not only provides a physical scaffold in which cells are embedded but also regulates many cellular processes, including growth, migration, differentiation, survival, homeostasis, and morphogenesis. Cells embedded in the ECM interact with this macromolecular network *via* their surface receptors, which include integrins, discoidin domain receptors, cell surface proteoglycans, and the hyaluronan receptor CD44 (67). During tumorigenesis, marked alterations take place in the ECM, leading to formation of a fibrotic stroma with increased stiffness, excessive deposition of ECM components, and release of proteolytic enzymes that result in abnormal ECM remodeling upon activation. These changes in the ECM further promote tumor progression and metastasis. The ECM has been identified to have an important role in the progression of residual cancer of HCC after RFA (68).

Zhang et al. (38) revealed that an increase in the ECM protein collagen I promotes progression of heat-exposed residual HCC cells, indicating the importance of collagen I in modulating residual HCC after incomplete heat treatment, and proposed that sorafenib could reverse collagen I-induced protumor effects. Zhang et al. (69) also showed that the increased matrix stiffness that occurs after RFA promoted proliferation, motility, and progression of heat-exposed residual HCC cells.

### 5.3 Tumor-associated macrophages

Macrophages play an important role in tumorigenesis. Regulation of the biological behavior of tumor cells by manipulation of the function of macrophages is a current focus in tumor research (70). Macrophages have strong plasticity, and their activated states and types have different effects on the biological behavior of tumors. Th1-activated macrophages (classical activation/M1-like) have anti-tumor activity and Th2-activated macrophages (bypass pathway/M2-like) are related to tumor growth and metastasis (71).

Tumor-associated macrophages (TAMs) are a major component of the tumor microenvironment and play pivotal roles in progression of HCC. Many studies have indicated that tumor-associated macrophages promote initiation, angiogenesis, and metastasis of tumors and suppression of adaptive immunity by production of a large number of cytokines, chemokines, growth factors and matrix metalloprotease in the tumor microenvironment (72). Collettini et al. (73) identified a large number of macrophages around the RFA area, which suggested that tumor-associated macrophages participate in progression of HCC after RFA. Rozenblum et al. (74) found that RFA induced large concentrations of macrophages around the necrotic area and that blockade of either IL-6 or c-met significantly reduced the proliferation of hepatocytes, with blockade of IL-6 reducing accumulation of both macrophages and myofibroblasts in the vicinity of the area of coagulation necrosis. Kumar et al. observed an increase in markers of tissue inflammation in the periablational rim and serum after hepatic RFA, including increased production of cytokines and recruitment of inflammatory cells (including macrophages, myofibroblasts, T-cells, and natural killer cells). Increased activation of COX-2 after hepatic RFA contributes to infiltration of periablational macrophages and inflammation-mediated distant tumor growth, which can be successfully suppressed with a COX-2 inhibitor (75).

### 5.4 Carcinoma-associated fibroblasts

Carcinoma-associated fibroblasts (CAFs) constitute a substantial proportion of the non-neoplastic mesenchymal cell compartment in various human tumors. These fibroblasts are phenotypically converted from their progenitors *via* interactions with nearby cancer cells during the course of tumor progression. The resulting CAFs, in turn, support the growth and progression of carcinoma cells. These fibroblasts have a major influence on the hallmarks of carcinoma and promote malignancy by secretion of tumor-promoting growth factors, cytokines, and exosomes, and by remodeling of the ECM (76).

Kumar et al. (75) observed an increase in alpha-smooth muscle actin (αSMA)-positive activated myofibroblasts after RFA. However, periablational recruitment of activated myofibroblasts was lower after daily exposure to celecoxib following RFA than after RFA alone. Rozenblum et al. (74) found that RFA induced a large accumulation of activated myofibroblasts around the necrotic zone. In addition to the accumulation of myofibroblasts, RFA induced proliferation of hepatocytes in both the ablated lobe and an untreated lobe, and blockade of either IL-6 or c-met significantly reduced global proliferation of hepatocytes. These changes, which were mediated *via* IL-6- and/or c-met, could have accounted for a proportion of the local and distant tumor recurrences observed after treatment. Ahmed et al. (77) demonstrated that the increase in heat shock protein induced by RFA could promote tumor growth and progression. Ma et al. (78) also showed that the gain-of-function p53 protein could bind selectively to the chaperone protein heat shock protein 90 and be packaged into small extracellular vesicles, which could be transferred to fibroblasts.

### 5.5 Hepatic stellate cells

CAFs in the liver are mainly derived from hepatic stellate cells (HSCs) (79). HSCs are liver sinusoidal resident vitamin A-storing cells and are considered to be the most relevant profibrogenic cell type operating in chronic liver diseases. During the process of liver injury, these cells undergo phenotypic transformation from ‘quiescent’ cells into ‘activated’ cells, which are characterized by proliferation, contractility, increased synthesis and secretion of ECM, altered matrix protease activity, and pro-mitogenic cytokines (80, 81). CAFs are composed of both fibroblasts and α-SMA-positive myofibroblasts, which are the hallmark of activated fibroblasts. Expression of α-SMA has long been regarded as the most reliable marker for detection of activated fibroblast populations in CAFs (82).

Zhang et al. (31) found that activated HSCs promoted progression of residual HCC cells after sublethal heat treatment from autophagic survival to proliferation *via* hepatocyte growth factor/c-Met signaling. A combined treatment regimen that included inhibition of autophagy and c-Met signaling could suppress progression of residual HCC after incomplete thermal ablation. Furthermore, Zhang et al. (83) demonstrated that activated HSCs can promote the stemness traits of residual HCC cells after incomplete thermal ablation and that metformin may be able to reverse this process. Zhang et al. (84) also showed that activated HSCs promoted progression of heat−treated residual HCC by release of POSTN, which could be inhibited by calcipotriol. Calcipotriol plus cisplatin could be used to thwart the accelerated progression of residual HCC after suboptimal heat treatment.

### 5.6 Platelets

Current research suggests that platelets have an important role in tumorigenesis, contributing to inflammation, angiogenesis and metastatic dissemination of tumor cells (85). In HCC, platelet activation is also an important risk factor for a poor postoperative prognosis (86). There is also research indicating that antiplatelet therapy reduces the risk of recurrence after surgical resection and improves overall survival in patients with HCC associated with viral hepatitis B (87).

Platelets can interact with adhesion molecules on the surface of endothelial cells and regulate the barrier function of these cells by releasing vesicles (88), which is an important step in the process of distant metastasis, and can increase vascular permeability. Kong et al. (63) found that ICAM-1 activates platelets in residual tumor tissue after RFA and promotes vascular permeability in TAECs *via* VE-cadherin and that anti-platelet and anti-ICAM-1 therapy could prevent progression of HCC after RFA. Furthermore, Jia et al. (66) compared the effect of platelet lysates in HCC cell lines before and after RFA and found that lysates obtained from patients after RFA of HCC could promote proliferation, migration, invasion, and VM of HCC cells. They also demonstrated that platelet lysates from patients who had undergone RFA accelerated metastasis of HCC cells to the lung.

**Figure 3:**
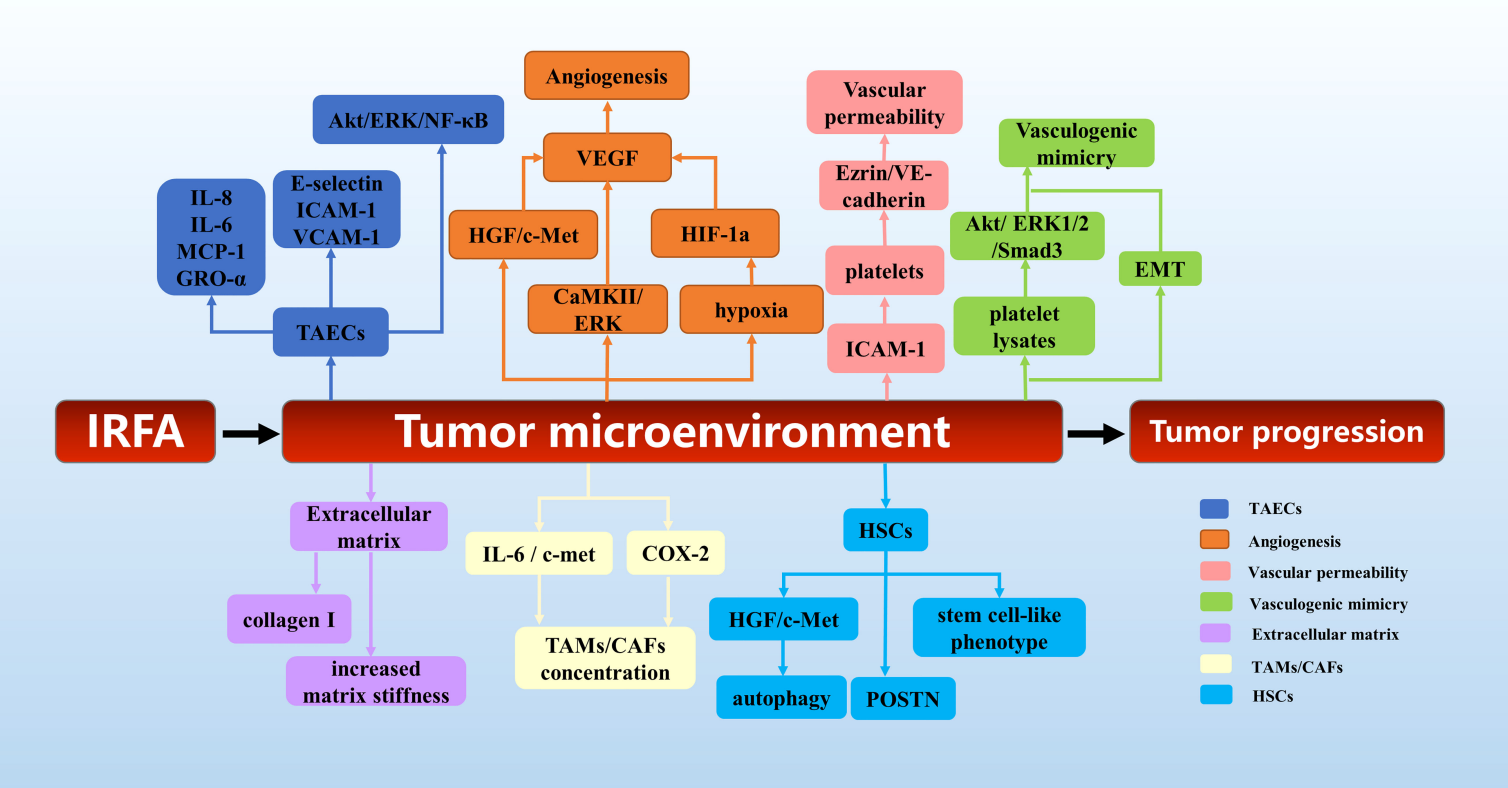
The mechanisms of tumor microenvironment in tumor progression after RFA of HCC.

In conclusion, as shown in **Figure 3**, the tumor microenvironment plays an important role in the tumor progression after IRFA of HCC. This provides a theoretical basis for the exploration of combined targeted therapy after RFA of HCC.

## 6 Strategies to prevent progression of HCC after RFA

Recent efforts have focused on multimodal management of HCC in which RFA is combined with other anti-cancer approaches to prevent progression of HCC after RFA.

### 6.1 Pathological complete ablation

In terms of pathology, HCC usually consists of the main tumor lesion, peritumoral microvascular invasion (MVI), and satellite lesions. In the late stage, portal vein tumor thrombus and extrahepatic metastasis can be found. Imaging can reveal the main tumor lesion and larger satellite lesions but not MVI and smaller satellite lesions. The extent of HCC that cannot be seen by imaging is usually much larger than that of the main lesion. Pathological complete ablation with no residual viable tumor cells requires complete ablation of all tumor tissue, including the main tumor lesion, peritumoral MVI, and satellite lesions, and is the most effective way of preventing disease progression (89).

In most cases, RFA completely ablates only the main HCC lesion, even if there is an ablation margin, what is obtained is usually the imaging complete ablation. The residual MVI and satellite lesion around the tumor will lead to tumor progression. The most effective way to achieve pathological complete ablation is to increase the ablation margin. Jiang et al. (90) demonstrated that the minimum ablation margin was significantly smaller for a tumor with local progression than for one without local progression. Li et al. (91) and Laimer et al. (92) also found that enlarging the ablation margin could significantly reduce the risk of tumor recurrence and improve the long-term survival rate. In a previous study, we demonstrated that repeated RFA with an ablation margin and transarterial chemoembolization improved the outcome in patients with large solitary HCCs measuring ≥10 cm (93). Moreover, our yet to be published current research demonstrates that long-term overall survival is not significantly different between anatomic resection and RFA with an ablation margin ≥1.0 cm in patients with a solitary HCC measuring ≤3 cm. Therefore, when performing RFA as a local ablation therapy to reduce the risk of local disease progression and improve the overall survival rate, we need to ablate not only the target tumor but also the apparently non-tumorous surrounding liver tissues, which could be harboring micrometastases and contain areas of microvascular invasion.

### 6.2 RFA plus targeted therapy or immunotherapy

In the past few years, a number of promising targeted therapies have emerged for HCC. Sorafenib is the classic molecular targeted agent and is approved by the US Food and Drug Administration (FDA) for the treatment of advanced HCC. When used to treat HCC, sorafenib is reported to activate several signaling pathways, in particular those for Raf/MEK/ERK, the VEGF receptor, and platelet-derived growth factor receptor. Sorafenib can also be used to prevent progression of HCC after RFA (94). Dong et al. (37) demonstrated that sorafenib suppressed EMT of HCC cells after inadequate RFA and could prevent progression of HCC after RFA. Kong et al. (44) showed that sorafenib could inhibit and prevent migration of a colony of HCC cells after RFA, that overexpression of IF1 could attenuate the effect of sorafenib in these cells, and that inhibition of IF1 could improve the therapeutic effect of sorafenib. Xu et al. (61) also found that sorafenib blocked the HIF-1α/VEGFA pathway, inhibiting tumor invasiveness and inducing apoptosis in hepatoma cells after RFA. Furthermore, Mertens et al. (95) demonstrated that sorafenib promoted necrosis after RFA, decreasing tissue repair and preventing disease progression. This reduction in tissue repair is caused by inhibition of neovascularization and reduced cell proliferation. Bevacizumab is the first anti-angiogenesis agent approved by the FDA. It is a humanized monoclonal antibody against VEGFA. One study in a rat model demonstrated that bevacizumab is useful for preventing rapid progression of residual HCC following RFA (60).

More recently, immunotherapy has emerged as the standard first-line treatment for patients with advanced HCC. Combination of RFA with cellular immunotherapy has attracted interest because of its synergistic anticancer effects and is expected to eradicate residual disease after RFA and prevent disease recurrence (96). Ma et al. (97) found that autologous RetroNectin-activated killer cells and suggested that adaptive immunotherapy might help to prevent recurrence of HCC after RFA. Kitahara et al. (98) also demonstrated that intratumoral injection of OK432-stimulated dendritic cells could prevent progression of residual HCC after RFA.

Although some studies have demonstrated that targeted therapy or immunotherapy after RFA provides better outcomes than RFA alone, more multicenter randomized clinical trials in large samples are needed to confirm the benefits of RFA plus targeted therapy or immunotherapy. Moreover, how to develop individualized treatment strategies to obtain the best treatment effect needs to be taken into consideration in clinical research.

### 6.3 RFA combined with other agents

Metformin is recommended as first-line therapy for all patients with a new diagnosis of type 2 diabetes mellitus. There are some epidemiologic data highlighting the positive effects of metformin on the incidence of cancer and its mortality. Metformin also appears to hold promise as a treatment for HCC. In one study, metformin was found to inhibit cell proliferation, invasion, and angiogenesis and to induce apoptosis in HCC (99). Zhang et al. (23) also found that metformin inhibited the growth of HCC cells after insufficient RFA and suggested that it could be used to prevent progression of HCC after RFA.

Arsenic trioxide (ATO) has been approved by the FDA as first-line treatment for acute promyelocytic leukemia (100). Recent *in vitro* studies have demonstrated that ATO can suppress HCC cells *via* various mechanisms, including suppression of proliferation, slowing invasion and migration, as well as reversing multidrug resistance (101–103). These effects suggest that ATO may be able to eradicate residual tumor cells. Dong et al. (104) demonstrated that ATO blocked the paracrine signaling of Ang-1 and Ang-2 by inhibiting p-Akt/Hif-1a and further suppressed angiogenesis of HCC after insufficient RFA. Chen et al. (105)also found that extensive angiogenesis after RFA could augment the enhanced permeability and retention effect and increase the enrichment of ATO-loaded ZIF-8 nanoparticles, which markedly inhibited residual tumor progression.

## 7 Conclusion

Various factors contribute to progression of residual HCC after RFA. Current research on the mechanisms of disease progression after RFA for HCC is mainly focused on changes in the biological behavior of tumor cells and remodeling of the tumor microenvironment. A number of studies performed in the clinical practice setting have confirmed that multimodal therapies that include RFA can indeed improve the outlook for patients with HCC. Further efforts are needed to optimize the protocol for each of the combination therapies and to establish the best combination strategy to prevent progression of HCC after RFA.

## Author contributions

JK and WS designed the manuscript. SLW, ZL, CYY, SD, JG, SK, RZ, SH, SHW, LX, CY performed literature research. SLW, ZL, CY drafted the article. JK and WS revised the article. All authors have read and approved the final article. All authors contributed to the article and approved the submitted version.

## Funding

This work was supported by Grant 82272760 from the National Natural Science Foundation of China; Grant QML20190306 from Beijing Hospitals Authority Youth Program; and Grant 7212044 from Beijing Natural Science Foundation.

## Conflict of interest

The authors declare that the research was conducted in the absence of any commercial or financial relationships that could be construed as a potential conflict of interest.

## Publisher’s note

All claims expressed in this article are solely those of the authors and do not necessarily represent those of their affiliated organizations, or those of the publisher, the editors and the reviewers. Any product that may be evaluated in this article, or claim that may be made by its manufacturer, is not guaranteed or endorsed by the publisher.
